# Coexistence of Fabry disease with IgM nephropathy: A case report

**DOI:** 10.1097/MD.0000000000017566

**Published:** 2019-10-11

**Authors:** Huizhen Wu, Tapas Ranjan Behera, Jianguang Gong, Quanquan Shen

**Affiliations:** a Department of Nephrology, Chun’an First People's Hospital, Hangzhou, Zhejiang, China; b Renal Division, Department of Medicine, Brigham and Women's Hospital, Harvard Medical School, Boston, MA; c Department of Nephrology, Zhejiang Provincial People's Hospital, People's Hospital of Hangzhou Medical College, Hangzhou, Zhejiang, China.

**Keywords:** Fabry disease, IgM nephropathy, lysosomal storage disorder

## Abstract

**Rationale:**

Coexistence of Fabry disease and IgM nephropathy is rare. The varying severity and unapparent clinical manifestation of Fabry disease makes it difficult to recognize when coexisting with another more prevalent cause of nephropathy requiring electron microscopy and genetic testing to confirm their coexistence.

**Patient concerns:**

A 54-year-old female presented with proteinuria without any clinical signs or family history of Fabry disease.

**Diagnoses:**

Immunostaining of the renal biopsy identified mesangial IgM deposition diagnosing it as IgM nephropathy. The light microscopy indicated prominent vacuolization of podocytes. Further examination of toluidine blue stained semi-thin sections and electron microscopy revealed blue bodies and myelin bodies in the cytoplasm of podocytes, respectively. Mutation analysis detected missense mutation establishing the diagnosis of coexisting Fabry disease.

**Interventions:**

The patient was treated with angiotensin-converting enzyme inhibitors. Enzyme replacement therapy was not administered due to financial constraints.

**Outcomes:**

After 2 months of treatment the patient demonstrated urine protein to creatinine ratio of 0.21 g/g.

**Lessons:**

Identifying coexistence of Fabry disease with other nephropathy requires meticulous pathologic investigations including electron microscopy especially when Fabry disease presents with atypical phenotype.

## 1 Introduction

Fabry disease is a rare, X-linked lysosomal storage disorder of glycosphingolipid catabolism resulting from a deficient activity of the lysosomal glycohydrolase α-galactosidase A (GLA).^[1]^ The enzyme is encoded by the *GLA* gene in the X chromosomal region Xq22.^[2]^ This enzymatic defect leads to progressive accumulation of globotriaosylceramide (GL-3) and related glycosphingolipids throughout the body. It is also known as Anderson–Fabry disease which was first described by Fabry and Anderson in 1898.^[3]^ Symptoms of the disease may appear since childhood, including angiokeratoma, neuropathic or limb pain (acroparesthesias), hypohidrosis (or hyperhidrosis), gastrointestinal symptoms, renal manifestations such as proteinuria, isosthenuria, polyuria and polydipsia; corneal and lenticular opacities, cardiovascular involvement including left ventricular hypertrophy and cerebrovascular involvement leading to wide range of neurologic symptoms may manifest. IgM nephropathy is predominantly an idiopathic glomerulonephritis characterized by the mesangial matrix expansion and presence of IgM located in the global and diffuse mesangial lesion. In this report, we present a case of the X-linked Fabry disease in a 54-year-old woman who showed atypical symptoms of the disease with coexistence of IgM nephropathy.

### 1.1 Consent statement

The patient provided informed consent for the publication of this case report and accompanying images. This study was performed in compliance with the Helsinki Declaration and was approved by the Institutional Review Board of Zhejiang Provincial People's Hospital.

## 2 Case report

A 54-year-old female was referred to nephrology department due to abnormal urinalysis. The patient was detected to have proteinuria in a screening examination 1 month ago. She had no complaints such as skin lesions, corneal opacity, neuropathic limb pain, temperature sensitivity, abnormal sweating, or gastrointestinal symptoms. She had medical history of type 2 diabetes mellitus diagnosed about 5 years ago for which she was being treated with gliclazide. Other than which she was not taking any medication, and did not have past exposure to medications such as chloroquine or amiodarone. The patient denied family history of any genetic disease including Fabry disease. Physical examination, electrocardiogram, ophthalmic examination, and blood tests including immunoglobulin and complement tests were done. A renal biopsy was performed to determine the reason of proteinuria. Two needle biopsy cores including about 25 glomeruli were submitted. Renal biopsy, with subsequent immunofluorescent, and electron microscopic interpretation were done. After obtaining the patient's informed consent, mutation analysis (Sanger sequencing and RFLP method) was performed.

Physical examination was unremarkable. Repeat urinalysis showed proteinuria (protein/creatinine 1.26 g/g). Kidney function was normal (blood urea nitrogen 5.51 mmol/L, creatinine 65.4 μmol/L), liver function, electrolytes, and complete blood count were within normal limits. Total protein and albumin were 64.6 and 40.4 g/L, respectively. Hemoglobin A1c was 5.9%. Immunology tests showed IgA and C3 level to be slightly decreased, 0.71 g/L (normal range 0.82–4.53 g/L) and 0.77 g/L (normal range 0.79–1.52 g/L), respectively. Other immunoglobulin and complement levels were normal. Electrocardiogram showed signs of left ventricular hypertrophy and ophthalmic examination revealed no abnormalities.

**Figure 1:**
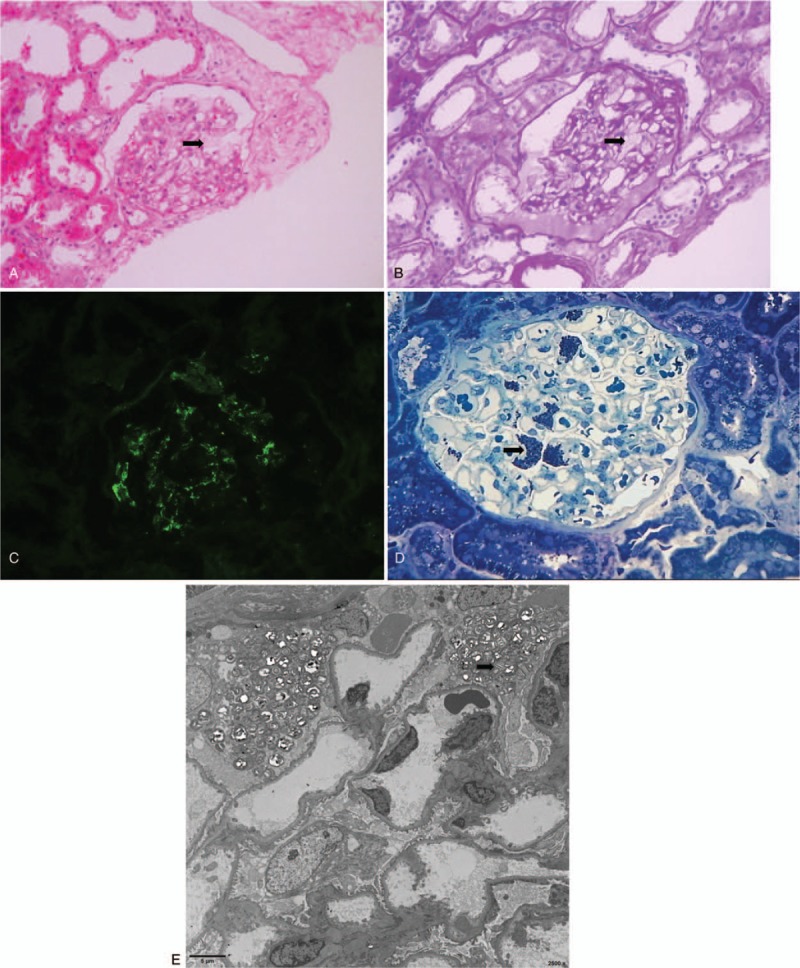
(A) Light microscopy showed remarkable vacuolization (black arrow) of podocytes (hematoxylin-eosin stain, ×200 magnification). (B) Light microscopy showed remarkable vacuolization (black arrow) of podocytes (periodic acid–schiff stain, ×200 magnification). (C) Immunofluorescent staining reveals moderate IgM staining at mesangial and capillary wall. (D) Examination of toluidine blue-stained semi-thin sections demonstrated blue bodies (black arrow) in the cytoplasm of podocytes (toluidine blue stain, ×400 magnification). (E) Electron microscopy shows prominent myelin bodies in podocyte cytoplasm (scale bar 5 μm).

Light microscopy showed hypercellularity and mesangial expansion in glomeruli and glomerular collapse with hyalinosis, other glomeruli contained strikingly enlarged and vacuolated podocytes (Fig. 1A, B). Vacuolated changes were also observed in some tubular epithelium cells (Fig. 1A, B). Immunofluorescence microscopy revealed 2+ granular IgM deposits in mesangial areas (Fig. 1C), while being negative for IgA, IgG, C3, C4, and C1q. Examination of toluidine blue-stained semi-thin sections and electron microscopy was performed, revealing blue bodies (Fig. 1D) and myelin-like bodies (Fig. 1E) in the cytoplasm of podocytes, respectively.

The mutation analysis identified a missense mutation c.902G > A (p.R301Q) in exon 6 (codon 301), which resulted due to the replacement of a glutamine for an arginine residue. Based on these findings, the patient was diagnosed with coexisting Fabry disease and IgM nephropathy. We decided to start therapy with angiotensin-converting enzyme inhibitors. Enzyme replacement therapy (ERT), however, was not administered due to financial constraints of the patient. After 2 months of treatment the patient demonstrated urine protein to creatinine ratio of 0.21 g/g.

## 3 Discussion

Fabry disease is a rare X-linked multiorgan disorder caused by mutations of the *GLA* gene that encodes alpha-galactosidase A, deficient activity of which results in the accumulation of globotriaosylceramide in lysosomes in multiple cell types throughout the body. However, due to unapparent clinical manifestation, biopsy, and genetic testing may be required to diagnose superimposed Fabry disease on other causes of nephropathy. Historically, females were considered to be asymptomatic carriers of the defective *GLA* gene; however, studies have shown that clinical manifestation in heterozygous females can vary widely from no apparent disease to full expression of disease.^[4]^ In the presented case, there were no apparent clinical symptoms or family history of Fabry disease, and as such it was impossible to detect it at an earlier stage.

The frequency of IgM nephropathy reported in renal biopsy series in adults in the literature has varied from 1.8% to 45%.^[5,6]^ This wide variation in the prevalence of IgM nephropathy may be due to varying biopsy indications, varying definitions used for the diagnosis, and genetic or environmental factors. Common biopsy indications in the cases diagnosed with IgM nephropathy are non-nephrotic proteinuria, nephrotic syndrome and hematuria. However, the most common presenting feature of IgM nephropathy is non-nephrotic proteinuria in adults.^[6]^ Our patient also had presented with non-nephrotic proteinuria.

The detection of a high level of autoantibodies has been found in patients with Fabry disease.^[7]^ However, specific link between IgM nephropathy and Fabry disease has not yet been identified. It has been suggested that glycosphingolipids accumulating in Fabry disease continuously and chronically stimulate the immune system and induce an autoimmune reaction.^[8]^ Thus, some patients with Fabry disease are prone to the development of IgM nephropathy which may be determined by different genetic and/or environmental factors. Considering the rare identification of IgM nephropathy with concurrent late onset Fabry disease, a missense mutation of p.R301Q as in this patient, may indicate possibility of the coexistence and may guide investigation appropriately. The patient's cardiac examination indicated left ventricular hypertrophy which is noted to be the most common cardiac manifestation in Fabry disease.^[9]^

Clinical trials have shown that recombinant human GLA replacement therapy can reverse substrate storage in the lysosomes. ERT should be initiated in all males with Fabry disease (including those with end-stage renal disease) and female carriers with substantial disease manifestations should be initiated as early as possible. However, no specific recommendation on optimal dosing is available. Because of the possibility of activation of immune system by sphingolipids in Fabry disease as evidenced by the high level of immunoglobulins in the patients of Fabry disease, the therapy recommendations need to be modulated for the associated IgM nephropathy.

The decision of choice of therapy in Fabry disease with associated IgM nephropathy need to be made with careful considerations including ERT and immunosuppressive therapy. Further trials need to be conducted in optimizing therapy especially in males with milder symptoms or carrier females with atypical late-onset symptoms. Because of the possible immunologic relationship between Fabry disease and IgM nephropathy with the given wide variability of the manifestation of Fabry disease, useful outcome measures for assessment of specific therapies need to be developed.

In conclusion, coexistence of Fabry disease with other nephropathies may remain undiagnosed because of its variable and nonspecific clinical manifestation. The detection of high level of autoantibodies in patients of Fabry disease with a possible cause being the chronic accumulation of glycosphingolipids stimulating the immune system may explain coexistence of Fabry disease in many other immunologic diseases. The identified mutation in this case may be a starting point to investigate an immunologic basis in the pathophysiology of coexistence of Fabry disease with IgM nephropathy. An increased vigilance for the clinicopathologic features may uncover cases of coexisting Fabry disease subdued by obvious clinical manifestation of other immunologic causes of nephropathy.

## Author contributions

**Conceptualization:** Huizhen Wu.

**Data curation:** Huizhen Wu, Jianguang Gong.

**Methodology:** Tapas Ranjan Behera, Jianguang Gong.

**Supervision:** Quanquan Shen.

**Writing – original draft:** Huizhen Wu.

**Writing – review & editing:** Tapas Ranjan Behera, Quanquan Shen.
